# Association of Social-Cognitive Factors with Individual Preventive Behaviors of COVID-19 among a Mixed-Sample of Older Adults from China and Germany

**DOI:** 10.3390/ijerph19116364

**Published:** 2022-05-24

**Authors:** Yanping Duan, Sonia Lippke, Wei Liang, Borui Shang, Franziska Maria Keller, Petra Wagner, Julien Steven Baker, Jiali He

**Affiliations:** 1 Department of Sport, Physical Education and Health, Hong Kong Baptist University, Hong Kong 999077, China; wliang1020@hkbu.edu.hk (W.L.); jsbaker@hkbu.edu.hk (J.S.B.); 2 Center for Health and Exercise Science Research, Hong Kong Baptist University, Hong Kong 999077, China; 3 Department of Health Sciences, Wuhan Institute of Physical Education, Wuhan 430079, China; h13163386915@163.com; 4 Department of Psychology & Methods, Jacobs University Bremen, 28759 Bremen, Germany; f.keller@jacobs-university.de; 5 Department of Social Sciences, Hebei Sport University, Shijiazhuang 050063, China; boruishang@hepec.edu.cn; 6 Institute for Exercise and Public Health, Leipzig University, 04109 Leipzig, Germany; petra.wagner@uni-leipzig.de

**Keywords:** COVID-19 pandemic, individual preventive behaviors, motivational and volitional factors, older adults, mixed sample

## Abstract

Identifying modifiable correlates of older adults’ preventive behaviors is contributable to the prevention of the COVID-19 and future pandemics. This study aimed to examine the associations of social-cognitive factors (motivational and volitional factors) with three preventive behaviors (hand washing, facemask wearing, and physical distancing) in a mixed sample of older adults from China and Germany and to evaluate the moderating effects of countries. A total of 578 older adults (356 Chinese and 222 German) completed the online cross-sectional study. The questionnaire included demographics, three preventive behaviors before and during the pandemic, motivational factors (health knowledge, attitude, subjective norm, risk perception, motivational self-efficacy (MSE), intention), and volitional factors (volitional self-efficacy (VSE), planning, and self-monitoring) of preventive behaviors. Results showed that most social-cognitive factors were associated with three behaviors with small-to-moderate effect sizes (*f*^2^ = 0.02 to 0.17), controlled for demographics and past behaviors. Country moderated five associations, including VSE and hand washing, self-monitoring and facemask wearing, MSE and physical distancing, VSE and physical distancing, and planning and physical distancing. Findings underline the generic importance of modifiable factors and give new insights to future intervention and policymaking. Country-related mechanisms should be considered when aiming to learn from other countries about the promotion of preventive behaviors.

## 1. Introduction

The novel coronavirus disease 2019 (COVID-19) pandemic has been the most severe global public health issue since December 2019, causing over 445 million confirmed cases and more than 6 million deaths worldwide inclusive of 10 March 2022 [1]. As a vulnerable population group, older adults suffered the most, accounting for nearly 75% of the COVID-19 relevant mortality globally [2,3]. Given that there is still not enough vaccination prevention for COVID-19 worldwide especially for older adults [4] and no guarantee for full protection from the pandemic even following vaccination [5], the everyday individual preventive actions, such as performing hand hygiene frequently, wearing facemasks, and keeping physical distancing in public areas play an important role in reducing the transmission of COVID-19 among older adults [6,7,8,9].

Public health organizations have been striving to develop behavioral interventions to promote individual preventive behaviors among the general population including older adults [10]. However, evidence has indicated that older adults showed a relatively low compliance with preventive behaviors compared to other age groups [11]. Identifying key correlates of the preventive behaviors that are potentially modifiable through intervention (e.g., social-cognitive factors), that can be targeted in messages or campaigns of behavioral intervention aimed at promoting preventive behaviors among older adults, is a recognized priority. 

In general, social-cognitive factors of behavior change comprise motivational factors associated with behavior initiation and volitional factors associated with behavior maintenance [12]. Recently, there has been an increasing group of evidence investigating preventive behaviors and their social-cognitive factors in older adults during the COVID-19 pandemic [13,14,15]. However, most of these studies focused only on the motivational factors of preventive behaviors while the volitional factors were comparably ignored. 

To maximize the prediction of social-cognitive factors towards COVID-19 preventive behaviors, a comprehensive review of these factors (motivational and volitional factors) is needed. The Theory of Planned Behavior (TPB), as a classic social-cognitive model identified specific motivational factors, including attitude (positive or negative evaluations towards the consequences of performing the intended behavior), subjective norm (perceived expectations of important others approving the intended behavior), perceived behavioral control (perception about being able to perform the intended behavior) and intention [16]. These factors have shown significant predictions related to hand washing [17,18] and facemask wearing [19]. In addition, previous research has shown that health knowledge was associated with hand washing behavior [20,21] and COVID-19 knowledge was correlated with preventive behaviors including wearing facemask and keeping physical distancing [22]. 

The Health Action Process Approach (HAPA), a widely used psychosocial model during the past two decades, suggests attention to critical factors not only in the motivational phase but also in the volitional phase [23]. During the motivational phase, risk perception (perceived susceptibility to a health threat in terms of both perceived vulnerability and perceived severity) and motivational self-efficacy (the beliefs about the ability to start the behavior even when facing difficulty) are considered important to form the intention of preventive behaviors (e.g., for facemask wearing [24]; for physical distancing [25]; for handwashing, facemask wearing, and physical distancing) [26]). After the intention is formed, self-regulatory strategies (e.g., planning, volitional self-efficacy, and self-monitoring) need to be enacted to ensure an intention is realized, and once initiated, maintained in the volitional phase. Particularly, planning includes action planning about “when”, “where,” and “how” to act as well as coping planning about how to overcome anticipated barriers to the action. Volitional self-efficacy contains beliefs about the capabilities to overcome barriers during the maintenance period and to regain control after a setback. Finally, self-monitoring adjusts behavior by monitoring when, where, and how long to perform the behavior. The prediction function of planning, volitional self-efficacy and self-monitoring is supported in hand washing and facemask wearing research [27,28]. 

As perceived behavior control (PBC) in TPB shares a synonymous construct with motivational self-efficacy in HAPA [29], the current study used motivational self-efficacy instead of PBC. After a review of the main social-cognitive factors (motivation and volition factors) of behaviors, this study adopted the motivational factors including attitude, subjective norm, motivational self-efficacy, risk perception, health knowledge, and intention, as well as the volitional factors including planning, volitional self-efficacy, and action control. 

It has been known that some preventive measures such as hand washing and facemask wearing diverge across Eastern and Western hemispheres [30,31,32]. Compared to people in Eastern countries (e.g., China, Japan, and South Korea), people in Western countries (e.g., Italy, UK, and USA) are more likely to wash hands frequently and less likely to wear facemasks in preventing the transmission of COVID-19 [31,32]. In addition, a cross-cultural study revealed that people in Europe had less knowledge of COVID-19 and were less aware of COVID-19 compared to people in Asia as the pandemic began to unfold in 2020 [33]. However, to the best of our knowledge, the impact of cross-cultural/country differences on the association between COVID-19 preventive behaviors and their social-cognitive factors (motivation and volition factors) has not been well explored to date. 

The novelty of this study is providing evidence of generically and country-specifically modifiable variables for promoting preventive behaviors among older adults from China and Germany during the peak global pandemic period in the middle of 2020. The study findings may provide information on public health approaches applied in these two countries in promoting preventive behavior among older adults during and beyond the COVID-19 pandemic. This study aimed to investigate: (1) the association of selected motivational factors and volitional factors with three preventive behaviors of older adults from China and Germany during the COVID-19 pandemic; and (2) the moderating effect of culture/country (China vs. Germany) on the associations of these social-cognitive factors with three preventive behaviors.

## 2. Materials and Methods

### 2.1. Study Design, Participants, and Procedure

This study adopted a cross-sectional design using an online questionnaire survey. To be eligible for inclusion, participants needed to meet the criteria, including: (1) aged 55 years or older; (2) have not been infected with the COVID-19; (3) have access to a mobile phone or laptop with internet connection; and (4) are able to read Chinese (for Chinese samples) or German (for German sample). Aiming to achieve a small-to-medium effect size (Cohen’s *f*^2^ = 0.085) on the association of social-cognitive factors with health behaviors [34], with an alpha of 0.05, a statistical power of 80%, and a response rate of 60% [35], a total of 335 participants were required.

Using a convenience sampling approach, Chinese participants were recruited from Wuhan, Hubei Province of China which was the most seriously infected region during the pandemic in 2020, while German participants were recruited from a national wide cohort. For Chinese samples, data collection started on 15 June 2020 and was completed on 10 July 2020. For the German samples, the duration of data collection was from 16 June 2020 to 17 February 2021. The survey was constructed and administered using online survey platforms (i.e., SOJUMP in China and Unipark in Germany). Recruitment information was disseminated by diverse channels, including mobile short message service, social media (e.g., WeChat, Weibo, QQ used in China; Twitter, Facebook used in Germany), personal networks, press releases, and network articles. All data were collected anonymously. Finally, we contacted 698 participants (434 Chinese and 264 German), among which 578 eligible participants (356 Chinese with mean age = 67.75 years, SD = 6.24; 222 German with mean age = 69.09 years, SD = 6.9) completed the online survey and were included in the analyses.

All participants in China and Germany were asked to sign an informed consent form on the first page of the survey platform before completing the questionnaires. Ethical approval for the study in China was obtained from the Research Ethics Committee of Hong Kong Baptist University (REC/19-20/0490). For the German study, ethical approval was obtained from the Ethics Committee of Jacobs University (Application Number: 2020_09). 

### 2.2. Measurement

A series of questionnaires were used to investigate older adults’ demographic information, preventive behaviors, and motivational and volitional factors of preventive behaviors. All questionnaires were adapted from well-established ones in previous studies and back-translated to Chinese and German by two independent bilingual translators. Each participant took 15–20 min to complete all online questionnaires. The questionnaire items and reliability are presented as follows:

#### 2.2.1. Demographic Information

The demographic characteristics included age, gender (male/female/other), marital status (single/married), country (China/Germany), living situation (alone/with children or spouse), education level (primary school or below/secondary school/university or above), occupational status (employed/unemployed), household income (below the average/average/above the average), children status (yes/no). Participants were also invited to report their chronic disease situation (yes/no), infected acquaintances (yes/no), perceived health status (bad/satisfactory/excellent), height (cm), and weight (kg). 

#### 2.2.2. Preventive Behaviors 

Preventive behaviors during the COVID-19 pandemic:

*Hand washing behavior* was measured using two items in accordance with the World Health Organization’s (WHO) recommendations (Cronbach’s α = 0.60). The frequency of hand washing behavior was evaluated with the stem “During the previous week, how frequently did you wash your hands with soap and water or alcohol-based hand rub (for at least 20 s, all surfaces of the hands)… ”, followed by two kinds of situations, i.e., “in the daily life situations (e.g., before preparing food; before eating; after defecation)” or “in disease-related situations (e.g., after blowing nose or sneezing; before and after caring for the sick)”. Older adults were asked to rate the two items on a 4-point Likert scale ranging from (1) never to (4) always. A higher average score of two items indicated performing better hand washing behavior. 

*Facemask wearing behavior* was measured with two items in accordance with the WHO recommendations (Cronbach’s α = 0.79). The questions were asked using the stem “During the previous week, I have usually worn a facemask properly…” followed by two different situations relevant to older adults, i.e., “when visiting public places (e.g., public transportation, supermarket)”, and “caring for a person with suspected COVID-19 infection”. Responses were scored on a 4-point Likert scale ranging from (1) strongly disagree to (4) strongly agree. A higher average score of two items indicated performing better facemask wearing behavior. As some of the items evaluated such as “caring for a person with suspected COVID-19 infection” may represent a rather situational context, a fifth answer category was provided for individuals who were not faced with such a situation termed as “not applicable”. In this case, only the score of option in daily life situation was used for analysis.

*Physical distancing behavior* was measured with two items according to the WHO recommendations (Cronbach’s α = 0.78). Participants were asked to assess their physical distancing behavior during the past week, with items such as (a) usually stayed out of crowded places or mass gatherings, and (b) usually kept space (at least 1.5 m) between myself and other people who are coughing or sneezing.” Answers were given on a 4-point Likert scale from (1) strongly disagree to (4) strongly agree. A higher average score of two items indicated better performing physical distancing behavior.

Past preventive behaviors before the COVID-19 pandemic:

Participants were asked to recall their three preventive behaviors before the pandemic of COVID-19 respectively. Items of each past preventive behavior were identical to those during the aforementioned COVID-19 pandemic. 

#### 2.2.3. Motivational Factors of Preventive Behaviors 

*Risk perception* was measured using one item for three preventive behaviors respectively, which was adapted from previous studies [36,37,38]. The participants were asked “Compared to an average person of your age and gender, what is your risk of COVID-19 infection from lack of frequent hand washing/facemask wearing/physical distancing?” with responses rated on a 6-point Likert scale from 1 = very low to 6 = very high. 

*Health knowledge* was measured using one item for three preventive behaviors respectively, which was adapted from previous studies [39,40,41]. The participants were asked “Have you known how and in what situations to wash hands/wear a facemask/keep a safe physical distancing in accordance with the WHO recommendations?” with responses rated on a 4-point scale with 1 (do not know), 2 (a little), 3 (most), and 4 (all). The higher score represented more sufficient health knowledge. 

*Attitude* was assessed using a common stem on three preventive behaviors. Such as “For me to wash hands frequently/wear a facemask/keep a safe physical distance during the outbreak of COVID-19 would be…” followed by two semantic differential items. Items were rated on a 6-point Likert scale: troubling-reassuring (1–6) and optional-necessary (1–6) [17,42,43]. A high total score means a positive attitude. The Cronbach alpha coefficient was 0.69 (China) and 0.75 (Germany) for hand washing behavior, 0.74 (China) and 0.77 (Germany) for mask wearing behavior and 0.80 (China) and 0.65 (Germany) for physical distancing behavior.

*Subjective norm* was assessed using one item measuring participants’ perceptions of important others’ approval on the three preventive behaviors [17,19,44]. The participants were asked “Most people who are important to me (e.g., my family members, friends, doctors) think that I should wear a facemask during the outbreak of COVID-19.” with responses rated on a 6-point Likert scale, from 1 = strongly disagree to 6 = strongly agree. 

*Intention* was assessed with one item for three preventive behaviors respectively, which was adapted from previous studies [45,46]. The participants were asked “Today and in the near future, I intend to frequently wash my hands in various situations (e.g., before eating, after going to the washroom, after blowing my nose or sneezing)” for hand washing, “Today and in the near future, I intend to properly wear a facemask in various situations (e.g., visiting public places)” for mask wearing behavior, and “Today and in the near future, I intend to keep a safe physical distance in various situations (e.g., staying out of crowded places or mass gatherings when I go outside of my home)” for physical distancing. Items were rated on a 6-point Likert scale, from 1 = strongly disagree to 6 = strongly agree. 

*Motivational self-efficacy* was assessed using one item measuring older adults’ level of confidence in starting to act on preventive behaviors. The participants were asked “I feel certain that I can begin to wash my hands frequently/wear a facemask/keep a safe physical distance, even if it would be difficult to change my routines.” Responses were rated on a 6-point Likert scale, from 1 = totally disagree to 6 = totally agree [24,45,47]. 

#### 2.2.4. Volitional Factors of Preventive Behaviors 

*Volitional self-efficacy* was assessed using one item measuring participants’ confidence of recovery of the behaviors, respectively. The participants were asked “I feel certain that I can restart to wash my hands frequently/wear face mask/keep a secure physical distance even if I forgot to do it a few times” with responses rated on a 6-point Likert scale, from 1 = totally disagree to 6 = totally agree [24,45,46]. 

*Planning* included action planning and coping planning. *Action planning* was assessed with one item for three preventive behaviors respectively. The items were “I have already made a concrete action plan for hand washing regarding when, where and how to…” followed by “wash my hands/wear face mask/keep a safe physical distance”. *Coping planning* was assessed by the item “I have made a coping plan to maintain frequent hand washing/mask wearing/physical distancing if I am confronted with some barriers”. Answers were given on a 6-point Likert scale from 1 = totally disagree to 6 = totally agree [8,19,45,46]. The Cronbach’s alpha coefficient was 0.75 (China) and 0.80 (Germany) for hand washing behavior, 0.84 (China) and 0.82 (Germany) for mask wearing behavior and 0.74 (China) and 0.83 (Germany) for physical distancing behavior.

*Self-monitoring* was assessed using one item measuring participants’ perceptions of their self-regulation over the preventive behaviors. The participants were asked “I have consistently monitored myself about how and in what situations to wash my hands/wear a face mask/keep a safe physical distance”, with responses rated on a 6-point Likert scale, from 1 = strongly disagree to 6 = strongly agree [12,18,48].

### 2.3. Statistical Analysis

Data analysis was conducted using IBM SPSS 26.0 (Armonk, NY, USA). Descriptive analyses including mean (standard deviation) and percentages used to present demographic differences between Chinese and German samples and were examined with independent t-test or chi-squared test. Moreover, the association of demographics and past preventive behaviors with the current three preventive behaviors were examined by *t*-tests, *F*-tests, and Pearson/Spearman *r* correlations. In addition, a series of univariate linear regressions were used to analyze the associations of social-cognitive factors with three preventive behaviors after control demographics and past behaviors. Furthermore, the moderating effect of the country on the association between social-cognitive factors and preventive behaviors was examined using multiple hierarchical linear regressions, where all independent variables were standardized using *Z* scores to avoid the collinearity problem. Particularly, significant demographic covariates and past behaviors identified in the primary analyses were added as predictors of each preventive behavior in Model 1. Significant motivational and volitional factors identified in the previous univariate linear regressions were sequentially added as predictors in Model 2 and Model 3. Afterwards, the binary dummy variable of country was added in the Model 4 and the interaction of country and social-cognitive predictors were added in the Model 5. To further elaborate the magnitude of the association between preventive behaviors and their associated factors in regression analyses, effect size (*f*^2^) was estimated with the conversion formula: *f*^2^ = *R*^2^/(1-*R*^2^), with 0.02, 0.15, and 0.35 indicating a small, medium, and large effect, respectively [12].

## 3. Results

### 3.1. Sample Characteristics 

As shown in Table 1, the sample in China was different from the sample in Germany concerning the majority of demographic variables, including age (mean age_China_ = 67.75 years vs. Mean age_Germany_ = 69.09 years; *t*_576_ = −2.41, *p* = 0.016), Body Mass Index (BMI) (BMI_China_ = 23.23 vs. BMI_Germany_ = 25.6; *t*_576_ = −7.72, *p* < 0.001), gender (females_China_ = 39.6% vs. females_Germany_ = 63.5%; *χ*^2^_1_ = 31.28, *p* < 0.001), marital status (single_China_ = 16.9% vs. single_Germany_ = 86.9%; *χ*^2^_1_ = 275.07, *p* < 0.001), education level (secondary school and above_China_ = 93.7% vs. secondary school and above_Germany_ = 84.3%; *χ*^2^_1_ = 21.8, *p* < 0.001), occupation status (unemployed_China_ = 98.6% vs. unemployed_Germany_ = 76.9%; *χ*^2^_1_ = 72.79, *p* < 0.001), household income (average and above_China_ = 79.8% vs. average and above_Germany_ = 89.7%; *χ*^2^_1_ = 70.78, *p* < 0.001), living situation (living with children/spouse_China_ = 91% vs. living with children/spouse_Germany_ = 68.5%; *χ*^2^_1_ = 47.81, *p* < 0.001), chronic disease (yes_China_ = 53.1% vs. yes_Germany_ = 42.8%; *χ*^2^_1_ = 5.57, *p* < 0.05), infected acquaintances (yes_China_ = 12.6%, yes_Germany_ = 37.4%; *χ*^2^_1_ = 51.96, *p* < 0.001), and perceived health status (satisfactory and above_China_ = 91.6% vs. satisfactory and above_Germany_ = 85.5%; *χ*^2^_1_ = 29.92, *p* < 0.001).

### 3.2. Descriptive Information of Study Variables 

As outlined in Table 2, for hand washing behavior (Mean = 3.35, SD = 0.6), there were significant differences in country (*t*_adjust_ = 10.78, *p* < 0.001), marital status (*t*_adjust_ = −7.88, *p* < 0.001), occupation status (*t* = 3.89, *p* < 0.001), household income (*F* = 3.09, *p* = 0.016), children status (*t* = 4.66, *p* < 0.001), living situation (*t*_adjust_ = −2.73, *p* = 0.007), and infected acquaintances (*t* = −3.79, *p* < 0.001). Hand washing was also significantly associated with age (*r* = −0.09, *p* = 0.025), BMI (*r* = −0.19, *p* < 0.001), and past behavior (*r* = 0. 49, *p* < 0.001). For facemask wearing behavior (Mean = 3.76, SD = 0.51) and physical distancing behavior (Mean = 3.64, SD = 0.48), no significant differences were found in demographic variables (all *p* > 0.05). Both behaviors were significantly correlated to the past behavior (*r*_facemask wearing_ = 0.09, *p* = 0.024; *r*_physical distancing_ = 0.29, *p* < 0.001). The descriptive information of motivational factors and volitional factors for each preventive behavior (mean value, SD) is also presented in Table 2.

### 3.3. Association of Motivational Factors, Volitional Factors with Three Preventive Behaviors

When controlling for significant demographics and past preventive behaviors, the associations of motivational factors and volitional factors with preventive behaviors in the univariate regression analysis are presented in the Supplementary Materials. Hand washing was significantly correlated to all motivational and volitional factors with small effect sizes (*f*^2^ = 0.02 to 0.08), except for the risk perception (*β* = 0.05, 95% CI = −0.01–0.05, *p* = 0.24). Facemask wearing was significantly correlated to all motivational and volitional factors with small effect sizes (*f*^2^ = 0.01 to 0.14), except for the health knowledge (*β* = 0.06, 95% CI = −0.01–0.07, *p* = 0.17). Physical distancing was significantly associated with all motivational and volitional factors with small-to-moderate effect sizes (*f*^2^ = 0.01 to 0.17).

### 3.4. Country Moderating the Associations of Social-Cognitive Factors with Three Preventive Behaviors

#### 3.4.1. Hand Washing Behavior

Except for country, significant demographic variables and past behaviors were first entered as independent variables in Model 1 (See Table 3). The linear combination of all aforementioned variables significantly predicted hand washing behavior (*R*^2^ = 0.33, *F*_(9, 559)_ = 29.64, *p* < 0.001). The significant motivational factors and volitional factors revealed in univariate analyses were entered in Model 2 and Model 3, respectively. In Model 2, only health knowledge, attitude, and intention significantly contributed to the model (*R*^2^ change = 0.11, *F*_(14, 559)_ = 29.57, *p* < 0.001), while in Model 3 only the planning significantly contributed to the model (*R*^2^ change = 0.02, *F*_(17, 559)_ = 26.53, *p* < 0.001). Country was entered in Model 4 and significantly contributed to this model (*R*^2^ change = 0.01, *F*_(18, 559)_ = 26.31, *p* < 0.001). Finally, the interactions between country and these social-cognitive factors were entered in Model 5. Terms for the interaction between country and volitional self-efficacy significantly contributed to the model (*R*^2^ change = 0.02, *F*_(26, 559)_ = 19.20, *p* < 0.001). The full model (Model 5) eventually accounted for 48% of variance in hand washing behavior. In addition, the effect size (*f*^2^) of association for each model showed that Model 1 *f*^2^ = 0.49, Model 2 *f*^2^ = 0.76, Model 3 *f*^2^ = 0.83, Model 4 *f*^2^ = 0.88, and Model 5 *f*^2^ = 0.94, suggesting the large effect of association (*f*^2^ > 0.35) was in all these models.

**Figure 1:**
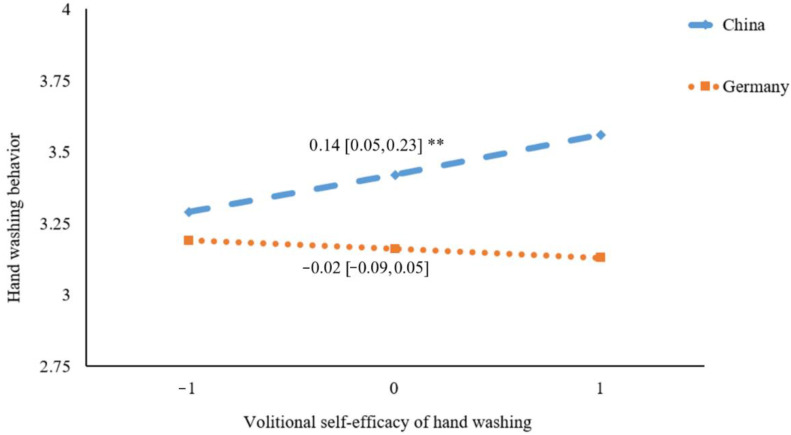
Plot of simple slopes showing the association between volitional self-efficacy and hand washing at different countries (*n* = 560); ** *p* < 0.01.

To further explore the interaction term, a simple slope analysis was employed to examine the moderating effect of the country on the associations of volitional self-efficacy with hand washing. As shown in Figure 1, there was no significant relationship between volitional self-efficacy and hand washing behavior in Germany (*β* = −0.02, 95% CI = [−0.09, 0.05], *p* = 0.54), whereas a significant positive correlation was found in China (*β* = 0.14, 95% CI = [0.05, 0.23], *p* = 0.002).

#### 3.4.2. Facemask Wearing Behavior

As all demographic variables were not significantly correlated to facemask wearing behavior in the aforementioned test, only past behavior as a covariate was added to Model 1 (see Table 4), where the linear model significantly predicted facemask wearing behavior (*R*^2^ = 0.01, *F*_(1, 577)_ = 5.12, *p* = 0.024). The significant motivational factors and volitional factors identified in univariate analyses were entered in Model 2 and Model 3, respectively. In Model 2, only motivational self-efficacy significantly contributed to the model (*R*^2^ change = 0.12, *F*_(6, 577)_ = 14.30, *p* < 0.001), while in Model 3 only volitional self-efficacy significantly contributed to this model (*R*^2^ change = 0.04, *F*_(9, 577)_ = 12.68, *p* < 0.001). Country was entered in Model 4 and significantly contributed to this model (*R*^2^ change = 0.05, *F*_(10, 577)_ = 15.40, *p* < 0.001). Finally, the interactions between country and these social-cognitive factors were entered in Model 5. Terms for the interaction between country and self-monitoring significantly contributed to this model (*R*^2^ change = 0.03, *F*_(18, 577)_ = 9.78, *p* < 0.001). The full model (Model 5) eventually accounted for 24% of variance in facemask wearing behavior. The effect size (*f*^2^) of factors associations for each model increased from a small level to a moderate level, with Model 1 *f*^2^ = 0.01, Model 2 *f*^2^ = 0.15, Model 3 *f*^2^ = 0.20, Model 4 *f*^2^ = 0.27, and Model 5 *f*^2^ = 0.31.

**Figure 2:**
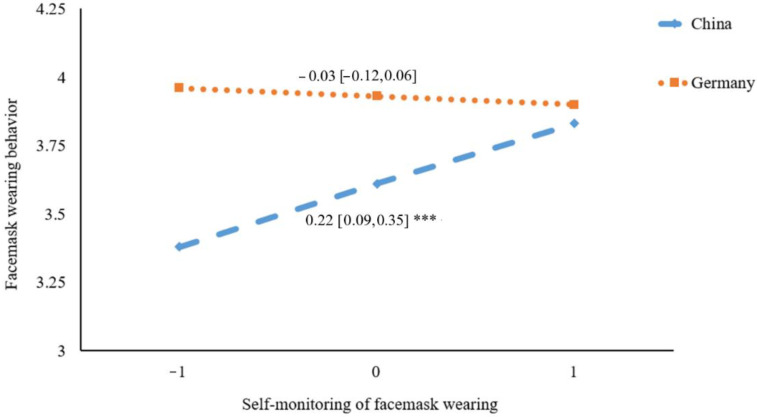
Plot of simple slopes showing the association between self-monitoring and facemask wearing at different countries (*n* = 578); *** *p* < 0.001.

Figure 2 presents the result of simple slope analysis. Results showed that there was a significant positive association between self-monitoring and facemask wearing behavior in China (*β* = 0.22, 95% CI = [0.09, 0.35], *p* < 0.001), whereas the association was not significant in Germany (*β* = −0.03, 95% CI = [−0.12, 0.06], *p* = 0.53).

#### 3.4.3. Physical Distancing Behavior

As all demographic variables were not significantly correlated to physical distancing behavior in the aforementioned test, only past behavior as a covariate was added to Model 1 (see Table 5), where the linear model significantly predicted physical distancing behavior (*R*^2^ = 0.08, *F*_(1, 577)_ = 53.02, *p <* 0.001). In Model 2 where the motivational factors were added, only intention significantly contributed to this model (*R*^2^ change = 0.13, *F*_(7, 577)_ = 21.20, *p* < 0.001). In Model 3, only volitional self-efficacy significantly contributed to the model (*R*^2^ change = 0.03, *F*_(10, 577)_ = 18.70, *p* < 0.001). Country was entered in Model 4 and significantly contributed to this model (*R*^2^ change = 0.08, *F*_(11, 577)_ = 24.80, *p* < 0.001). Finally, for the interaction terms, country x motivational self-efficacy, country x volitional self-efficacy, and country x planning significantly contributed to the Model 5 (*R*^2^ change = 0.02, *F*_(20, 577)_ = 14.77, *p* < 0.001). The full model (Model 5) accounted for 35% of variance in physical distancing behavior. Results indicated a small effect size of factor associations for Model 1 (*f*^2^ = 0.09), a moderate effect size for Model 2 and 3 (*f*^2^ = 0.27 and 0.33), and a large effect size for Model 4 and 5 (*f*^2^ = 0.48 and 0.53).

**Figure 3:**
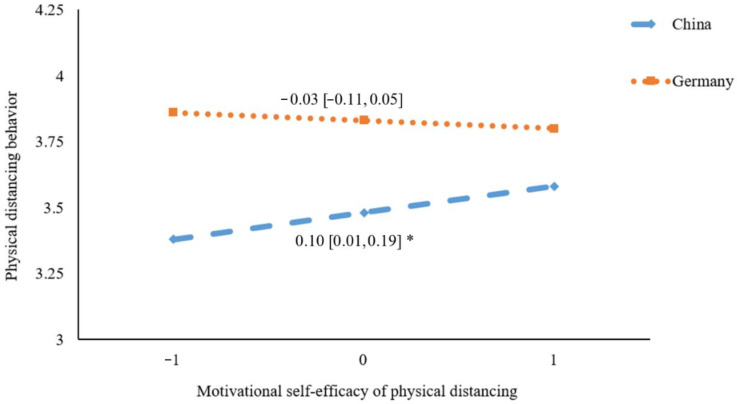
Plot of simple slopes showing the association between motivational self-efficacy and physical distancing at different countries (*n* = 578); * *p* < 0.05.

**Figure 4:**
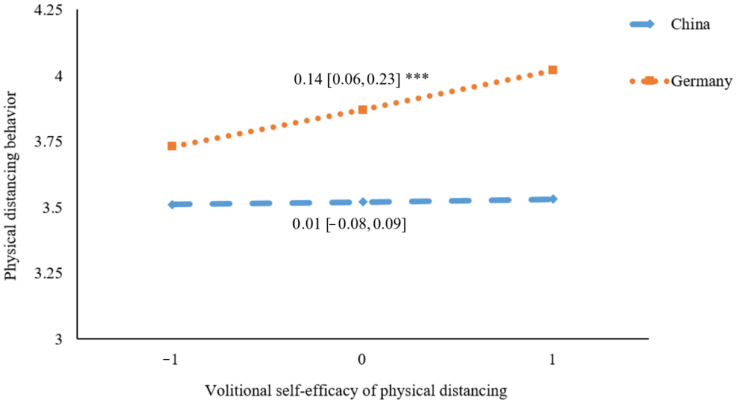
Plot of simple slopes showing the association between volitional self-efficacy and physical distancing at different countries (*n* = 578); *** *p* < 0.001.

**Figure 5:**
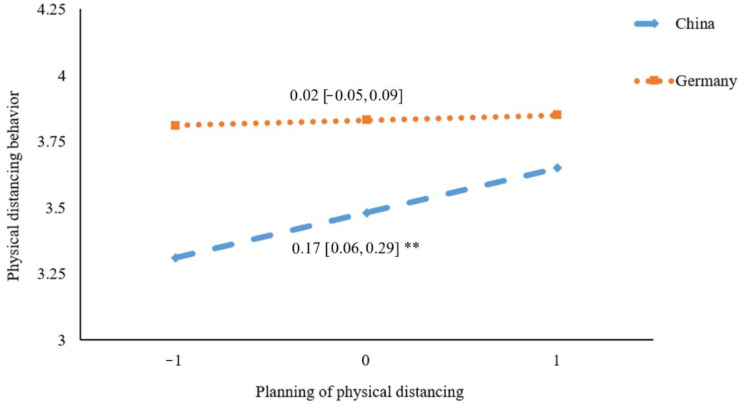
Plot of simple slopes showing the association between planning and social distancing at different countries (*n* = 578); ** *p* < 0.01.

Figure 3, Figure 4 and Figure 5 present the results of simple slope analyses. It was revealed that the association between motivational self-efficacy and physical distancing was not significant in Germany (*β* = −0.03, 95% CI = [−0.11, 0.05], *p* = 0.51), whereas a significant positive association was found in China (*β* = 0.10, 95% CI = [0.01, 0.19], *p* = 0.03) (see Figure 3). For volitional self-efficacy, it was found to be significantly and positively correlated to physical distancing behavior in Germany (*β* = 0.14, 95% CI = [0.06, 0.23], *p* < 0.001), whereas the association was not significant in China (*β* = 0.01, 95% CI = [−0.08, 0.09], *p* = 0.84) (see Figure 4). For planning, a significant positive association with physical distancing was found in China (*β* = 0.17, 95% CI = [0.06, 0.29], *p* = 0.004), but was not found in Germany (*β* = 0.02, 95% CI = [−0.05, 0.09], *p* = 0.56) (see Figure 5).

## 4. Discussion

This study was conducted to examine the association of theory-based social-cognitive factors with preventive behaviors of older adults and to evaluate the moderating effect of culture/country on the associations of factors with preventive behaviors of the elderly. Findings of the current study revealed differences in three preventive behaviors in older adults between China and Germany. This was noted even after controlling for demographic, motivational, and volitional factors: While older adults in China performed more hand washing behavior, older adults in Germany performed more facemask wearing behavior and physical distancing in public spaces. 

Without controlling for demographics, all recorded motivational and volitional factors were significantly associated with the three behaviors: As motivational factors, health knowledge, attitude, subjective norm, risk perception, motivational self-efficacy, and intention were imperative for performing the behaviors. In addition, the volitional factors such as volitional self-efficacy, planning and self-monitoring also revealed importance in predicting preventive behaviors in the mixed sample. These results are in line with previous findings [16,18,19,49]. As a result of these findings, previously identified key correlates of the preventive behaviors are suitable targets for intervention, that is, they can be addressed with messages or campaigns of behavioral intervention aimed at promoting preventive behaviors [9]. 

When taking demographic factors into account to predict hand washing behavior, past behavior, health knowledge, intention and planning remained significantly associated, which is consistent with previous studies [28,49,50,51,52]. Remarkably, when also taking the country into account, demographic factors were not significantly interrelated with the behavioral outcome variables anymore. This underlines that differences between the two countries cannot be attributed to demographic factors but rather to cultural differences. Empirical support was found that an interaction of country and volitional self-efficacy exists underlining the moderating effect of culture/country (China vs. Germany) on the associations of these factors for hand washing behavior and that attitude as well as volitional self-efficacy could explain additional variance. In brief, when designing hand washing behavioral interventions in China and Germany, past behavior, health knowledge, attitude, volitional self-efficacy, and planning should be addressed and in China even further attention should be put on increasing volitional self-efficacy. 

When investigating facemask wearing, past behavior, motivational self-efficacy, and volitional self-efficacy were salient predictors without controlling for country. Interestingly, intention and planning were not significant determinants to facemask wearing behavior in the current study, which is partially line with previous studies [28,51]. The discrepancy may be attributable to the different sample characteristics (e.g., young adults vs. older adults) and data collection period (during lock-down vs. after lock-down). When investigating the potential moderating role of the country it turned out that there was only interaction with self-monitoring: While facemask wearing could not be further increased with more self-monitoring in Germany, in China self-monitoring was related to more facemask wearing behavior. 

Regarding physical distancing, past behavior, health knowledge, and volitional self-efficacy remained significant also when taking the country into account. Country was not only a moderator for motivational self-efficacy but also for volitional self-efficacy and planning. Importantly, past behavior, health knowledge, intention, and planning also remained with their main effects on behavior independently of country underlining their generic importance: Only if individuals know why and how to perform physical distancing, and if they have performed this before and intend to repeat it, then they are more likely to also adopt and maintain this behavior. This finding is partially in line with a recent review study [53], which indicated that the population who were more likely to comply with social distancing included those who had high levels of knowledge about social distancing and had high intention level to perform it. In contrast, self-efficacy needs to be addressed in a country-specific manner: while in China, more motivational self-efficacy is imperative for more preventive physical distancing, in Germany more volitional self-efficacy seems to be required. This might be interpreted by the cultural difference (e.g., collectivism vs. individualism) [54,55]. For collectivism-oriented countries (e.g., China), the networks and physical connection are commonly highlighted. Chinese older adults may be not used to or not confident to keep physical distancing from their family and friends. Therefore, motivational self-efficacy is needed to facilitate the initiation process of physical distancing behavior among them. In contrast, German older adults under individualism-oriented cultural context may be confident to perform social distancing but need volitional self-efficacy more to maintain their physical distancing behavior. However, this assumption has not been systemically examined in the current study, which warrants further research.

The findings of this study fill the evidence gap because no such cross-cultural study was published prior to testing these motivational and volitional factors for all three preventive behaviors of older adults. In conclusion, regarding the research questions, this study presents unique findings: (1) the association of selected motivational factors and volitional factors with three preventive behaviors of older adults from China and Germany during the COVID-19 pandemic is not only generic for some factors but also specific for others. (2) The moderating effect of culture/country (China vs. Germany) on the associations of these factors with three preventive behaviors revealed importance especially for motivational self-efficacy (China-physical distancing), volitional self-efficacy (China-hand washing and Germany-physical distancing) as well as self-monitoring (China-facemask wearing). This should be considered when aiming to learn from other countries: aiming to improve physical distancing in Germany in the same way as in China may not work as different self-efficacy beliefs need to be addressed (motivational in China and volitional in Germany). Remarkably, risk perception was not imperative although most campaigns aim to make individuals more aware of the risks. In contrast, health knowledge and the other motivational and volitional factors are more important, underlining the importance of mastery (past behavior) and communicating own controllability in terms of intention, planning, and self-efficacy.

Several limitations should be noted. Firstly, the cross-sectional design limits the ability to infer causal relationships. Applying longitudinal designs or experimental tests is needed. Secondly, due to the limited resource available during the pandemic, we applied a convenience sampling approach to recruit older adults who are more educated and technology savvy from two countries. This may lead to the sampling bias and weaken the generalizability of samples. Future studies should employ random sampling approaches with inclusion of more countries and larger sample size to enhance the representativeness and generalizability. Furthermore, all the variables were measured by self-reported scales which might cause response bias (e.g., recall bias, multiple entries, random answers, and social desirability). In addition, considering the parsimonious mode and operational feasibility of online survey among older adults, some variables were measured using one or two items. Although the validity and reliability of these items were approved in previous studies [8,12,18], the measure biases cannot be ignored and applying comprehensive questionnaires to measure relevant outcomes is warranted.

## 5. Conclusions

In conclusion, the findings of the current study can guide the design of more effective messages or campaigns: Not only does health knowledge need to be addressed but importantly the factors more closely correlated with behavior change such as intention, planning and self-efficacy. While country-related mechanisms seem to exist, findings underline the generic importance of modifiable factors and addressing them through intervention especially in a resource-oriented way communicating the need and controllability of the behaviors. As older people are especially vulnerable to develop a severe disease, enabling them to protect themselves is key in the long term. We should also help this population to develop intentions and plans and strengthen their self-efficacy.

## Figures and Tables

**Table 1:** Characteristics of overall sample and by country.

	Overall (*n* = 578)	China (*n* = 356)	Germany (*n* = 222)	*χ*^2^/*t*	*d*	*p*
**Age, mean (SD)**	68.27 (6.53)	67.75 (6.24)	69.09 (6.9)	−2.41	0.21	0.016
**BMI, mean (SD)**	24.14 (3.76)	23.23 (2.95)	25.6 (4.4)	−7.72	0.66	<0.001
**Gender, *n* (%)**				31.28	0.54	<0.001
Female	282 (48.8%)	141 (39.6%)	141 (63.5%)			
Male	296 (51.2%)	215 (60.4%)	81 (36.5%)			
**Marital status, *n* (%)**				275.07	1.94	<0.001
Single	253 (43.8%)	60 (16.9%)	193 (86.9%)			
Married/partnered	324 (56.1%)	296 (83.1%)	28 (12.6%)			
Missing	1 (0.2%)	0	1 (0.4%)			
**Education level, *n* (%)**				21.8	0.22	<0.001
Primary school or below	38 (6.6%)	20 (5.6%)	18 (8.1%)			
Secondary school	203 (35.1%)	154 (43.2%)	49 (22.1%)			
University or above	318 (55%)	180 (50.5%)	138 (62.2%)			
Missing	19 (3.3%)	2 (0.7%)	17 (7.6%)			
**Occupation status, *n* (%)**				72.79	1.68	<0.001
Employed/working	54 (9.3%)	5 (1.4%)	49 (23.1%)			
Unemployed	514 (88.9%)	351 (98.6%)	163 (76.9%)			
Missing	10 (1.8%)	0	10 (4.5%)			
**Household income, *n* (%)**				70.78	0.68	<0.001
Below the average	94 (16.3%)	72 (20.2%)	22 (10.3%)			
Average	296 (51.2%)	217 (61.0%)	79 (37.1%)			
Above the average	179 (31.0%)	67 (18.8%)	112 (52.6%)			
Missing	9 (1.5%)	0	9 (4.0%)			
Children status *, *n* (%)				NA	NA	NA
Yes (have children)	521 (90.1%)	354 (99.4%)	167 (75.2%)			
No	57 (9.9%)	2 (0.6%)	57 (25.7%)			
**Living situation, *n* (%)**				47.81	0.85	<0.001
Living alone	102 (17.6%)	32 (9%)	70 (31.5%)			
Living with children/spouse	476 (82.4)	324 (91%)	152 (68.5%)			
**Chronic disease, *n* (%)**				5.57	0.22	0.018
Yes	284 (49.1%)	189 (53.1%)	95 (42.8%)			
No	293 (50.7%)	167 (46.9%)	126 (56.8%)			
Missing	1 (0.2%)	0	1 (0.4%)			
**Infected acquaintances, *n* (%)**				51.96	0.81	<0.001
Yes	128 (22.1%)	45 (12.6%)	83 (37.4%)			
No	443 (76.6%)	311 (87.4%)	132 (59.5%)			
Missing	7 (1.3%)	0	7 (3.1%)			
**Perceived health status, *n* (%)**				29.92	0.45	<0.001
Bad	62 (10.7%)	30 (8.4%)	32 (14.5%)			
Satisfactory	277 (48.0%)	148 (41.6%)	129 (58.4%)			
Excellent	238 (41.2%)	178 (50.0%)	60 (27.1%)			
Missing	1 (0.2%)	0	1 (0.4%)			

* It is not applicable to conduct χ^2^ test as the number in one cell of Chinese data is less than 5.

**Table 2:** Descriptive statistics for the associations of demographic information, past behaviors with three preventive behaviors, as well as motivational factors and volitional factors in the total sample (*n* = 560–578).

	Hand Washing		Facemask Wearing		Physical Distancing	
	Mean (SD)	*F/t/r*	Mean (SD)	*F/t/r*	Mean (SD)	*F/t/r*
**Total**	3.35 (0.6)		3.76 (0.51)		3.64 (0.48)	
**Country**						
China	3.55 (0.51)	10.78 ^a^ ***	3.73 (0.56)	−1.63	3.62 (0.48)	−0.87
Germany	3.02 (0.6)		3.80 (0.48)		3.66 (0.49)	
**Age**		−0.09 *		0.01		−0.03
**Gender**						
Female	3.30 (0.59)	−1.87	3.76 (0.49)	0.18	3.67 (0.47)	1.29
Male	3.39 (0.61)		3.76 (0.53)		3.61 (0.49)	
**Marital status**						
Single	3.13 (0.63)	−7.88 ^a^ ***	3.76 (0.53)	0.08	3.63 (0.49)	−0.44
Married	3.52 (0.52)		3.76 (0.5)		3.64 (0.47)	
**Education level**						
Primary school or below	3.25 (0.68)	2.94	3.78 (0.40)	2.72	3.59 (0.53)	1.87
Secondary school	3.43 (0.57)		3.70 (0.56)		3.60 (0.50)	
University or above	3.31 (0.62)		3.80 (0.48)		3.67 (0.44)	
**Occupation status**						
Employed	3.05 (0.62)	3.89 ***	3.75 (0.52)	0.09	3.60 (0.56)	0.58
Unemployed	3.38 (0.60)		3.76 (0.46)		3.64 (0.47)	
**Household income**						
Below the average	3.34 (0.63)	4.27 *	3.66 (0.53)	2.87	3.57 (0.47)	2.04
Average	3.41 (0.56)		3.75 (0.56)		3.63 (0.51)	
Above the average	3.25 (0.65)		3.76 (0.51)		3.69 (0.44)	
**Children status**						
Yes (have children)	3.39 (0.59)	4.66 ***	3.76 (0.50)	−0.28	3.64 (0.47)	−0.73
No	3.00 (0.58)		3.78 (0.57)		3.68 (0.54)	
**Living situation**						
Living alone	3.19 (0.67)	−2.73 ^a^ *	3.66 (0.66)	−1.84	3.68 (0.49)	0.84
Living with children/spouse	3.38 (0.58)		3.78 (0.47)		3.63 (0.48)	
**Chronic disease**						
Yes	3.38 (0.61)	1.32	3.75 (0.55)	−0.3	3.66 (0.48)	1.01
No	3.31 (0.60)		3.77 (0.47)		3.62 (0.49)	
**Infected acquaintances**						
Yes	3.17 (0.61)	−3.86 ***	3.75 (0.60)	−0.32	3.59 (0.50)	−1.31
No	3.40 (0.59)		3.77 (0.48)		3.65 (0.48)	
**Perceived health status**		2.33		0.75		1.50
Bad	3.26 (0.69)		3.81 (0.50)		3.71 (0.42)	
Satisfactory	3.32 (0.63)		3.77 (0.55)		3.66 (0.49)	
Excellent	3.41 (0.55)		3.73 (0.47)		3.60 (0.48)	
BMI		−0.19 ***		0.05		−0.02
Past behavior	2.94 (0.70)	0.49 ***	2.53 (1.16)	0.09 *	2.87 (0.90)	0.29 ***
**Motivational factors**						
Health knowledge	4.22 (0.70)		3.69 (0.99)		3.65 (0.99)	
Attitude	5.47 (0.94)		5.50 (1.02)		5.62 (0.88)	
Subjective norm	5.64 (0.85)		5.66 (0.90)		5.68 (0.80)	
Risk perception	4.06 (1.66)		4.89 (1.32)		4.85 (1.35)	
Motivational self-efficacy	5.60 (0.92)		5.76 (0.76)		5.50 (0.95)	
Intention	5.67 (0.76)		5.67 (0.95)		5.68 (0.79)	
**Volitional factors**						
Volitional self-efficacy	5.59 (0.95)		5.73 (0.85)		5.63 (0.84)	
Planning	5.35 (1.16)		5.59 (0.96)		5.47 (0.97)	
Self-monitoring	5.31 (1.22)		5.66 (0.89)		5.44 (1.07)	

^a^ adjusted estimated for unbalanced groups; *** *p* < 0.001; * *p* < 0.05.

**Table 3:** Multiple hierarchical regression results for prediction of hand washing behavior (*n* = 560).

Variables	Model 1		Model 2		Model 3		Model 4		Model 5	
	*B* [95% CI]	*β*	*B* [95% CI]	*β*	*B* [95% CI]	*β*	*B* [95% CI]	*β*	*B* [95% CI]	*β*
Age	−0.002 [−0.01, 0.01]	−0.02	−0.001 [−0.01, 0.01]	−0.01	<0.001 [−0.01, 0.01]	−0.003	<0.001 [−0.01. 0.01]	−0.002	0.001 [−0.01, 0.01]	0.01
Marital status	0.23 [0.13, 0.33]	0.19 ***	0.20 [0.11, 0.29]	0.16 ***	0.18 [0.09, 0.27]	0.15 ***	0.08 [−0.03, 0.18]	0.06	0.07 [−0.04, 0.17]	0.05
Occupation	−0.15 [−0.31, 0.01]	−0.07	−0.11 [−0.26, 0.04]	−0.05	−0.10 [−0.25, 0.05]	−0.05	−0.03 [−0.18, 0.12]	−0.02	−0.02 [−0.17, 0.13]	−0.01
Household income	0.01 [−0.06, 0.07]	0.01	−0.01 [−0.07, 0.05]	−0.01	−0.01 [−0.07, 0.06]	−0.01	0.02 [−0.04, 0.08]	0.02	0.02 [−0.04, 0.08]	0.02
Children status	−0.14 [−0.30, 0.01]	−0.07	−0.12 [−0.26, 0.03]	−0.06	−0.10 [−0.24, 0.04]	−0.05	−0.04 [−0.19, 0.10]	−0.02	−0.05 [−0.19, 0.10]	−0.02
Living situation	0.06 [−0.06, 0.17]	0.04	0.03 [−0.07, 0.14]	0.02	0.01 [−0.10, 0.12]	0.01	−0.03 [−0.14, 0.08]	−0.02	−0.03 [−0.14, 0.08]	−0.02
Infected acquaintances	−0.001 [−0.11, 0.11]	−0.001	0.003 [−0.10, 0.11]	0.002	0.003 [−0.10, 0.10]	0.002	0.001 [−0.10, 0.10]	<0.001	−0.002 [−0.10, 0.10]	−0.002
BMI	−0.02 [−0.03, −0.004]	−0.10 **	−0.01 [−0.02, −0.003]	−0.08 *	−0.01 [−0.02, −0.002]	−0.08 *	−0.01 [−0.02, 0.001]	−0.06	−0.01 [−0.02, 0.002]	−0.05
Past behavior	0.37 [0.31, 0.43]	0.43 ***	0.23 [0.17, 0.29]	0.27 ***	0.22 [0.15, 0.28]	0.25 ***	0.21 [0.14, 0.27]	0.24 ***	0.19 [0.13, 0.26]	0.22 ***
Health knowledge			0.08 [0.04, 0.12]	0.13 ***	0.08 [0.04, 0.12]	0.13 ***	0.10 [0.06, 0.14]	0.17 ***	0.10 [0.06, 0.15]	0.17 ***
Subjective norm			0.05 [−0.01, 0.10]	0.08	0.03 [−0.02, 0.09]	0.05	0.02 [−0.03, 0.07]	0.03	−0.01 [−0.08, 0.06]	−0.02
Attitude			0.08 [0.03, 0.13]	0.13 **	0.05 [−0.002, 0.10]	0.08	0.04 [−0.01, 0.09]	0.07	0.06 [0.01, 0.11]	0.10 *
Motivational self-efficacy			−0.001 [−0.05, 0.05]	−0.001	−0.03 [−0.08, 0.02]	−0.05	−0.03 [−0.08, 0.03]	−0.04	−0.02 [−0.08, 0.03]	−0.04
Intention			0.12 [0.07, 0.17]	0.20 ***	0.06 [0.001, 0.12]	0.10 *	0.06 [0.004, 0.12]	0.10 *	0.03 [−0.03, 0.10]	0.06
Volitional self-efficacy					0.04 [−0.02, 0.09]	0.06	0.04 [−0.02, 0.09]	0.06	0.08 [0.02, 0.14]	0.13 *
Planning					0.11 [0.04, 0.18]	0.18 **	0.10 [0.03, 0.16]	0.16 **	0.13 [0.03, 0.22]	0.21 **
Self-monitoring					0.02 [−0.04, 0.09]	0.04	0.02 [−0.05, 0.08]	0.03	0.02 [−0.05, 0.09]	0.03
Country							−0.12 [−0.19, −0.06]	−0.20 ***	−0.13 [−0.20, −0.06]	−0.22 ***
Country × Health knowledge									0.01 [−0.03, 0.06]	0.02
Country × Subjective norm									0.05 [−0.01, 0.11]	0.10
Country × Attitude									−0.02 [−0.07, 0.03]	−0.04
Country × Motivational self-efficacy									0.01 [−0.04, 0.06]	0.01
Country × Intention									0.05 [−0.01, 0.10]	0.08
Country × Volitional self-efficacy									−0.08 [−0.13, −0.02]	−0.15 **
Country × Planning									−0.03 [−0.11, 0.05]	−0.06
Country × Self-monitoring									−0.02 [−0.08, 0.05]	−0.03

Marital status: 0 = single; 1 = partnered; Occupation: 0 = unemployed, 1 = (self-)employed/working; Household income: 0 = below average; 1 = average; 2 = above average; Children status: 0 = no children, 1 = one or more child; Living situation: 0 living alone, 1 = living with children/spouse; Infected acquaintances: 0 = no infected acquaintances; 1 = infected acquaintances. *** *p* < 0.001; ** *p* < 0.01; * *p* < 0.05.

**Table 4:** Multiple hierarchical regression results for prediction of facemask wearing behavior (*n* = 578).

Variables	Model 1		Model 2		Model 3		Model 4		Model 5	
	*B* [95% CI]	*β*	*B* [95% CI]	*β*	*B* [95% CI]	*β*	*B* [95% CI]	*β*	*B* [95% CI]	*β*
Past behavior	0.04 [0.01, 0.08]	0.09 *	0.003 [−0.03, 0.04]	0.04	0.003 [−0.03, 0.04]	0.01	0.09 [0.05, 0.14]	0.20 ***	0.08 [0.04, 0.13]	0.19 ***
Risk perception			−0.01 [−0.06, 0.03]	−0.03	−0.02 [−0.06, 0.03]	−0.03	−0.01 [−0.05, 0.04]	−0.01	−0.01 [−0.06, 0.06]	−0.01
Subjective norm			0.02 [−0.04, 0.08]	0.04	−0.003 [−0.07, 0.06]	−0.01	0.02 [−0.04, 0.09]	0.05	−0.01 [−0.09, 0.07]	−0.02
Attitude			0.01 [−0.05, 0.07]	0.02	−0.01 [−0.07, 0.05]	−0.02	0.01 [−0.05, 0.06]	0.01	0.004 [−0.06, 0.06]	0.01
Motivational self-efficacy			0.15 [0.08, 0.22]	0.30 ***	0.10 [0.02, 0.17]	0.19 **	0.08 [0.01, 0.15]	0.16 *	0.11 [0.02, 0.19]	0.21 *
Intention			0.02 [−0.03, 0.07]	0.04	−0.03 [−0.08, 0.03]	−0.06	−0.01 [−0.06, 0.05]	−0.02	−0.02 [−0.10, 0.05]	−0.04
Volitional self-efficacy					0.07 [.01, 0.13]	0.14 *	0.05 [−0.01, 0.11]	0.10	0.03 [−0.03, 0.09]	0.06
Planning					0.04 [−0.03, 0.11]	0.08	0.04 [−0.04, 0.11]	0.07	0.06 [−0.05, 0.16]	0.11
Self-monitoring					0.07 [−0.004, 0.15]	0.14	0.07 [−0.003, 0.14]	0.14	0.13 [0.04, 0.21]	0.25 **
Country							0.16 [0.11, 0.22]	0.32 ***	0.16 [0.10, 21]	0.31 ***
Country × Risk perception									0.003 [−0.04, 0.05]	0.01
Country × Subjective norm									0.05 [−0.02, 0.12]	0.11
Country × Attitude									0.02 [−0.04, 0.07]	0.03
Country × Motivational self-efficacy									−0.03 [−0.10, 0.05]	−0.06
Country × Intention									<0.001 [−0.06, 0.06]	−0.001
Country × Volitional self-efficacy									0.05 [−0.01, 0.11]	0.12
Country × Planning									−0.01 [0.09, 0.08]	−0.02
Country × Self-monitoring									−0.12 [−0.20, −0.05]	−0.28 **

*** *p* < 0.001; ** *p* < 0.01; * *p* < 0.05.

**Table 5:** Multiple hierarchical regression results for prediction of physical distancing behavior (*n* = 578).

Variables	Model 1		Model 2		Model 3		Model 4		Model 5	
	*B* [95% CI]	*β*	*B* [95% CI]	*β*	*B* [95% CI]	*β*	*B* [95% CI]	*β*	*B* [95% CI]	*β*
Past behavior	0.16 [0.11, 0.20]	0.29 ***	0.06 [0.01, 0.11]	0.11 *	0.06 [0.02, 0.11]	0.12 **	0.10 [0.06, 0.14]	0.19 ***	0.09 [0.05, 0.13]	0.17 ***
Health knowledge			0.02 [−0.02, 0.06]	0.04	0.02 [−0.02, 0.06]	0.05	0.07 [0.03, 0.11]	0.14 ***	0.08 [0.04, 0.12]	0.16 ***
Risk perception			−0.01 [−0.05, 0.03]	−0.01	−0.01 [−0.05, 0.03]	−0.02	0.02 [−0.02, 0.06]	0.04	0.02 [−0.02, 0.05]	0.03
Subjective norm			0.02 [−0.02, 0.07]	0.05	0.01 [−0.03, 0.06]	0.03	0.02 [−0.02, 0.07]	0.04	0.01 [−0.04, 0.06]	0.01
Attitude			0.04 [−0.01, 0.09]	0.08	0.02 [−0.03, 0.07]	0.04	0.01 [−0.04, 0.05]	0.01	−0.01 [−0.06, 0.04]	−0.02
Motivational self-efficacy			0.02 [−0.03, 0.08]	0.05	−0.03 [−0.09, 0.03]	−0.05	0.02 [−0.03, 0.08]	0.05	0.05 [−0.01, 0.12]	0.11
Intention			0.13 [0.08, 0.18]	0.28 ***	0.07 [0.02, 0.13]	0.14 *	0.05 [−0.01, 0.1]	0.10	0.08 [0.01, 0.16]	0.17 **
Volitional self-efficacy					0.13 [0.07, 0.18]	0.26 ***	0.10 [0.04, 0.15]	0.19 ***	0.06 [−0.001, 0.12]	0.13
Planning					0.05 [−0.01, 0.11]	0.10	0.06 [−0.001, 0.12]	0.12	0.12 [0.04, 0.19]	0.24 **
Self-monitoring					−0.02 [−0.08, 0.04]	−0.04	0.01 [−0.04, 0.07]	0.03	−0.02 [−0.11, 0.08]	−0.04
Country							0.17 [0.13, 0.22]	0.36 ***	0.17 [0.13, 0.22]	0.36 ***
Country × Health knowledge									−0.03 [−0.07, 0.004]	−0.07
Country × Risk perception									0.004 [−0.03, 0.04]	0.01
Country × Subjective norm									0.02 [−0.03, 0.06]	0.04
Country × Attitude									0.04 [−0.01, 0.08]	0.08
Country × Motivational self-efficacy									−0.06 [−0.12, −0.003]	−0.14 *
Country × Intention									0.04 [−0.11, 0.02]	−0.10
Country × Volitional self-efficacy									0.07 [0.01, 0.12]	0.15 *
Country × Planning									−0.07 [−0.14, −0.01]	−0.16 *
Country × Self-monitoring									0.04 [−0.04, 0.12]	0.09

*** *p* < 0.001; ** *p* < 0.01; * *p* < 0.05.

## Data Availability

Requests of data and materials should be directed to the study directors: Yanping Duan (duanyp@hkbu.edu.hk) and/or Sonia Lippke (s.lippke@jacobs-university.de).

## Supplementary Materials

The following supporting information can be downloaded at: https://www.mdpi.com/article/10.3390/ijerph19116364/s1, Table S1. Results of univariate regressions between motivational factors, volitional factors and preventive behaviors (*n* = 560–578).
