# Supplementary material for: Association of Social-Cognitive Factors with Individual Preventive Behaviors of COVID-19 among a Mixed-Sample of Older Adults from China and Germany
Source: Int J Environ Res Public Health. 2022 May 24;19(11):6364. doi: 10.3390/ijerph19116364 (PMC9180272; doi:10.3390/ijerph19116364)
Supplement: Supplementary file 1 [file ijerph-19-06364-s001.zip › ijerph-1727249-supplementary.pdf]

Table S1. Results of univariate regressions between motivational factors, volitional factors and preventive behaviors (n = 560–578).

	Hand Washing		Facemask Wearing		Physical Distancing	
	**B [95%CI]**	***β* (*f*²)**	**B [95%CI]**	***β* (*f*²)**	**B [95%CI]**	***β* (*f*²)**
**Motivational factors**						
Health knowledge	0.16 [0.10, 0.22]	0.18 (0.03) ***	−0.002 [−0.05, 0.05]	−0.003 (0.003)	0.06 [0.01, 0.10]	0.11 (0.01) *
Attitude	0.15 [0.10, 0.19]	0.23 (0.04) ***	0.13 [0.08, 0.17]	0.25 (0.06) ***	0.15 [0.10, 0.19]	0.27 (0.07) ***
Subjective norm	0.14 [0.09, 0.19]	0.19 (0.03) ***	0.16 [0.11, 0.21]	0.28 (0.08) ***	0.14 [0.09, 0.19]	0.23 (0.05) ***
Risk perception	0.02 [−0.01, 0.05]	0.05 (0.002)	0.05 [0.01, 0.08]	0.12 (0.01) **	0.03 [0.004, 0.06]	0.09 (0.01) *
Motivational self-efficacy	0.11 [0.06, 0.15]	0.17 (0.02) ***	0.24 [0.19, 0.29]	0.35 (0.14) ***	0.15 [0.11, 0.19]	0.29 (0.08) ***
Intention	0.21 [0.16, 0.27]	0.27 (0.06) ***	0.14 [0.09, 0.18]	0.25 (0.06) ***	0.22 [0.18, 0.27]	0.37 (0.13) ***
**Volitional factors**						
Volitional self-efficacy	0.14 [0.10, 0.19]	0.23 (0.05) ***	0.21 [0.16, 0.26]	0.36 (0.14) ***	0.23 [0.19, 0.28]	0.40 (0.17) ***
Planning	0.17 [0.13, 0.21]	0.32 (0.08) ***	0.18 [0.14, 0.22]	0.34 (0.11) ***	0.16 [0.12, 0.20]	0.33 (0.10) ***
Self-monitoring	0.13 [0.09, 0.16]	0.26 (0.05) ***	0.21 [0.16, 0.25]	0.36 (0.14) ***	0.12 [0.08, 0.16]	0.27 (0.06) ***

*** $p < 0.001$; ** $p < 0.01$; * $p < 0.05$.
